# The role of the speech and language therapist in the management of dysphagia in monkeypox

**DOI:** 10.1590/2317-1782/20232022241

**Published:** 2023-07-10

**Authors:** Thales Rafael Correia de Melo Lima, Brenda Carla Lima Araújo, Paulo Ricardo Martins-Filho

**Affiliations:** 1 Laboratório de Patologia Investigativa, Universidade Federal de Sergipe – UFS - Aracaju (SE), Brasil.; 2 Programa de Pós-graduação em Ciências da Saúde, Universidade Federal de Sergipe – UFS - Aracaju (SE), Brasil.; 3 Departamento de Fonoaudiologia, Universidade Federal de Sergipe – UFS - Aracaju (SE), Brasil.

Dear Editor,

Monkeypox is a double-stranded DNA virus of the Poxviridae family that has spread globally since May 2022, posing a serious public health threat. The primary human-to-human transmission routes include respiratory droplets and close contact with skin lesions^1^. The majority of cases in the current outbreak have occurred in men who have sex with men (MSM), and the lesions are most commonly found in the anogenital region. Some patients have also presented with oral and oropharyngeal lesions, which can progress to painful ulcers and tonsillar abscesses^2^. Furthermore, dysphagia has been shown in approximately 25% of monkeypox patients requiring hospital admission^3^.

Because dysphagia can be found in patients with the most severe forms of monkeypox^4^, it is critical that speech and language therapists are prepared to identify swallowing difficulties early and provide appropriate treatment for each case, avoiding dysphagia-associated complications such as dehydration, malnutrition, aspiration pneumonia, and prolonged hospital stay. Patients with oropharyngeal lesions must be evaluated using specific anamnesis, validated questionnaires, and clinical swallowing examination. It is also necessary to inspect and manipulate oral structures, as well as evaluate pain and masticatory function. Furthermore, due to disease transmission pathways, speech and language therapists must care for monkeypox patients in accordance with biosafety best practices.

Videofluoroscopic swallowing study (VFSS) is recognized as the gold-standard technique to assess dysphagia and to determine the most appropriate treatment plan for patients with swallowing disturbances. However, flexible endoscopic evaluation of swallowing (FEES) can be used as an alternative method when VFSS was either not available or not applicable. FEES can easily and directly assess oropharyngeal secretion management and the efficacy of cleaning mechanisms such as coughing and throat clearing^5^. Therefore, we recommend that patients with monkeypox and oropharyngeal involvement be evaluated by a multidisciplinary team, including (1) an oral diet assessment with multiple consistencies using disposable utensils; (2) cranial nerve evaluation; and (3) adoption of clinically appropriate compensatory strategies to determine the safest and most adequate nutritional route, considering medical history, current disease, and oropharyngeal performance.

In this letter, we highlight important considerations on the role of the speech and language therapist in the diagnosis and management of dysphagia in monkeypox patients. Studies on the functional consequences of the disease are needed to understand possible sequelae and specific needs of these patients.
